# Involvement of M1 Macrophage Polarization in Endosomal Toll-Like Receptors Activated Psoriatic Inflammation

**DOI:** 10.1155/2018/3523642

**Published:** 2018-12-16

**Authors:** Chih-Hao Lu, Chao-Yang Lai, Da-Wei Yeh, Yi-Ling Liu, Yu-Wen Su, Li-Chung Hsu, Chung-Hsing Chang, S.-L. Catherine Jin, Tsung-Hsien Chuang

**Affiliations:** ^1^Immunology Research Center, National Health Research Institutes, Miaoli, Taiwan; ^2^Department of Life Sciences, National Central University, Zhongli District, Taoyuan, Taiwan; ^3^Institute of Molecular Medicine, College of Medicine, National Taiwan University, Taipei, Taiwan; ^4^Skin Institute, Hualien Tzu Chi Hospital, Hualien, Taiwan; ^5^Institute of Medical Sciences, Tzu Chi University, Hualien, Taiwan; ^6^Program in Environmental and Occupational Medicine, Kaohsiung Medical University, Kaohsiung, Taiwan

## Abstract

Psoriasis is a chronic inflammatory skin disorder that affects ~2%–3% of the worldwide population. Inappropriate and excessive activation of endosomal Toll-like receptors 7, 8, and 9 (TLRs 7–9) at the psoriatic site has been shown to play a pathogenic role in the onset of psoriasis. Macrophage is a major inflammatory cell type that can be differentiated into phenotypes M1 and M2. M1 macrophages produce proinflammatory cytokines, and M2 macrophages produce anti-inflammatory cytokines. The balance between these two types of macrophages determines the progression of various inflammatory diseases; however, whether macrophage polarization plays a role in psoriatic inflammation activated by endosomal TLRs has not been investigated. In this study, we investigated the function and mechanism of macrophages related to the pathogenic role of TLRs 7–9 in the progression of psoriasis. Analysis of clinical data in database revealed significantly increased expression of macrophage markers and inflammatory cytokines in psoriatic tissues over those in normal tissues. In animal studies, depletion of macrophages in mice ameliorated imiquimod, a TLR 7 agonist-induced psoriatic response. Imiquimod induced expression of genes and cytokines that are signature of M1 macrophage in the psoriatic lesions. In addition, treatment with this TLR 7 agonist shifted macrophages in the psoriatic lesions to a higher M1/M2 ratio. Both of the exogenous and endogenous TLR 7–9 ligands activated M1 macrophage polarization. M1 macrophages expressed higher levels of proinflammatory cytokines and TLRs 7–9 than M2 macrophages. These results suggest that by rendering macrophages into a more inflammatory status and capable of response to their ligands in the psoriatic sites, TLR 7–9 activation drives them to participate in endosomal TLR-activated psoriatic inflammation, resulting in an amplified inflammatory response. Our results also suggest that blocking M1 macrophage polarization could be a strategy which enables inhibition of psoriatic inflammation activated by these TLRs.

## 1. Introduction

Psoriasis is a chronic inflammatory skin disease that affects 2%–3% of the worldwide population. The disease is associated with red, scaly, and raised plaques that are the result of a marked thickening of the epidermis induced by enhanced keratinocyte proliferation, leukocyte infiltrates, and inflammation. This disease can be caused by many genetic and external factors, such as immune disorders, skin injuries, microbial infections, environmental influences, weather, and stress, and has a big effect on the quality of life of the patients. Although fairly widespread, the molecular mechanisms underlying the pathogenesis of this disease are not fully understood [1–4].

Ten Toll-like receptors (TLRs) are identified in humans [5]. Of the 10 human TLRs, TLR 1, TLR 2, TLR 4, TLR 5, and TLR 6 are expressed on the cell surface. TLR 3, TLR 7, TLR 8, and TLR 9 are localized to intracellular vesicles, including endosomes, and are referred to as endosomal TLRs [6–9]. A recent study showed that TLR 10 is detectable on the cell surface but is more abundant intracellularly [10]. These TLRs belong to a family of pattern recognition receptors that are expressed in innate immune cells for the detection of microbial pathogen-associated molecular patterns, including lipoprotein, zymosan, lipopolysaccharide (LPS), flagellin, and microbial nucleic acids, and for the initiation of host immune responses [6–9]. In addition, these TLRs are activated by damage-associated molecular patterns (DAMPs), which are endogenously released by activated or necrotic cells and molecules in the extracellular matrix that are upregulated or degraded following tissue damage [11–13]. Activation of TLRs causes the expression of inflammatory genes to mediate the host's responses against microbial infection and tissue repair; however, excessive inflammation that results from activation of these TLRs has also been suggested to play a key role in the pathogenesis of inflammatory diseases, including atherosclerosis, cancer, rheumatoid arthritis, systemic lupus erythematosus, and psoriasis [14–17].

Macrophages originate from circulating monocyte precursors and extravagate into target tissues, where they become dependent on the microenvironment and differentiate into mature macrophages that polarize into different subsets. Two major subsets of these are the classically activated M1 macrophages and the alternatively activated M2 macrophages. M1 polarization is driven by the Th1 cytokine interferon-*γ* (IFN-*γ*) and microbial products, such as LPS. In contrast, M2 macrophages are polarized by different stimuli, such as macrophage colony-stimulating factor (M-CSF), interleukin-4 (IL-4), IL-10, and IL-13 [18, 19]. M1 macrophages are involved in inflammatory responses by producing chemokine ligands, such as chemokine (C-X-C motif) ligands 1–3 (CXCL1–3), CXCL5, and CXCL8–10, and proinflammatory cytokines, such as tumor necrosis factor- (TNF-) *α*, IL-1, IL-6, and IL-12, and type I interferons (IFN) for immune stimulation and defense against microbial infections. On the other hand, M2 macrophages are associated with anti-inflammatory responses and influence tissue repair by generating anti-inflammatory cytokines, such as IL-10 [20–22]. The M1 and M2 phenotypes represent two extreme ends of a continuum of intermediate phenotypes. Macrophages are actually very diverse and plastic. Even after a macrophage has adopted a phenotype, it is still able to change in response to stimulation from its environment [23]. Because chemokines and cytokines are major mediators of tissue injury or damage, a balance between M1 and M2 macrophages can regulate the initiation, progression, and cessation of inflammatory diseases [24–27].

Much progress has been made in recent years to understand endosomal TLRs, particularly the TLR 7-, TLR 8-, and TLR 9- (TLR 7–9-) mediated pathogenesis of psoriasis. The current model of the mechanism of their role indicates that microbial infections or skin injuries trigger the secretion of the cathelicidin antimicrobial peptide (LL37) from keratinocytes and the release of self-DNA and self-RNA from dead cells. LL37 forms a complex with these self-nucleic acids to activate TLRs 7–9 in dendritic cells (DCs), which results in the production of various proinflammatory cytokines that further activate other cell types, such as T cells and keratinocytes, and generate chronic psoriatic inflammation [28–33]. Nevertheless, although a macrophage is a major inflammatory cell type, whether the pathogenic role of endosomal TLRs in psoriasis involves their activation and polarization is not clear [31–33]. In this study, we investigated the function and mechanism of macrophages in TLR 7- to TLR 9-mediated psoriatic inflammation.

## 2. Materials and Methods

### 2.1. Reagents and Antibodies

Thiostrepton and azithromycin were purchased from Sigma-Aldrich (St. Louis, MO, USA). TLR ligands, including Pam3Cys, polyinosinic-polycytidylic acid (polyI:C), LPS, and R848, were purchased from InvivoGen (San Diego, CA, USA). CpG-2006 was purchased from Invitrogen (Carlsbad, CA, USA) or Genomics BioSci & Tech (New Taipei City, Taiwan). LL37 was purchased from GeneDireX (Gueishan Township, Taiwan). Human recombinant IFN-*γ* and IL-4 were purchased from PeproTech (Rocky Hill, NJ, USA).

### 2.2. Bioinformatics Analysis of Gene Expression in Patients with Psoriasis

Gene Expression Omnibus (GEO) allows user query, to download experiments, and to analyze gene expression profiles following its instruction (https://www.ncbi.nlm.nih.gov/geo/). GEO databases (https://www.ncbi.nlm.nih.gov/gds/) were searched for expression profiles of macrophage marker genes in normal and psoriatic tissues from patients.

### 2.3. Animal Studies

Animal experiments were approved by the Institutional Animal Care and Use Committee of the National Health Research Institutes (NHRI), Miaoli, Taiwan. Balb/c mice were maintained and handled in accordance with the stated guidelines.

### 2.4. Cell Culture and Bone Marrow-Derived Macrophage Production

THP-1 cells, a line of human monocytic cells derived from an acute monocytic leukemia patient, were grown in Roswell Park Memorial Institute 1640 (RPMI) medium supplemented with 10% fetal bovine serum (FBS). Bone marrow-derived macrophages (BMDMs) were from the bone marrow cells isolated from 6- to 8-week-old mice. These cells were cultured in Dulbecco's modified Eagle's medium (DMEM) and L929-conditioned medium at a 7 : 3 ratio and supplemented with 10% FBS for 5 d to generate BMDMs. BMDMs were then grown in DMEM supplemented with 10% FBS.

### 2.5. Activation and Polarization of the Monocytic THP-1 Cells into M1 and M2 Macrophages

THP-1 monocytes were activated into macrophages using 100 ng/mL phorbol-12-myristate-13-acetate (PMA) (Calbiochem, Temecula, CA, USA) for 24 h and then washed with medium. The medium was changed every other day for 6 d before polarization. The THP-1 macrophages were then polarized using 20 ng/mL IFN-*γ* for the M1 macrophages and 20 ng/mL IL-4 for the M2 macrophages. To study macrophage polarization induced by different TLR ligands, such as R848, CpG-2006, LL37/DNA, and LL37/RNA, the THP-1 macrophages were stimulated with the TLR ligand, as indicated, and analyzed by RT-qPCR for signature gene expression that would indicate M1 and M2 macrophages.

### 2.6. Analysis Using Real-Time Quantitative Polymerase Chain Reaction

Total RNA was purified using TRIzol (Invitrogen, Carlsbad, CA, USA) according to the manufacturer's protocols. Reverse transcription (RT) was performed using the SuperScript III first-strand synthesis system (Invitrogen, Carlsbad, CA, USA) and oligo-dT for first-strand cDNA synthesis. Real-time quantitative polymerase chain reaction (RT-qPCR) was conducted using the ABI PRISM 7900*HT* sequence detection system (Applied Biosystems Inc., Foster City, CA, USA) and KAPA SYBR fast qPCR kit (Sigma-Aldrich, St. Louis, MO, USA) for gene expression analysis. Data were analyzed using the 2^−ΔΔCt^ method described in the ABI user manual. The expression of mRNA was normalized to that of glyceraldehyde 3-phosphate dehydrogenase (GAPDH), and the data were expressed as fold expression relative to the mRNA with the lowest expression. Sequences of primers for amplification of human genes are shown in Supplementary Table S1; for amplification of mouse genes, sequences of primers are shown in Supplementary Table S2.

### 2.7. Cytotoxicity Assay

The cytotoxicity of different compounds was analyzed using the CellTiter 96 AQueous Non-Radioactive Cell Proliferation (MTS) Assay (Promega, Madison, WI, USA) following the manufacturer's protocol. Briefly, the cells were treated with various concentrations of thiostrepton or azithromycin as indicated for 24 h. MTS solution was added to each well. After 2 h, the absorbance at 490 nm was measured using an EnVision Multilabel Plate Reader (PerkinElmer, Waltham, MA, USA).

### 2.8. Enzyme-Linked Immunosorbent Assay for Cytokine Production

Macrophages were treated with or without different reagents as indicated for 24 h. The cell culture media were collected for measurement of cytokine productions using enzyme-linked immunosorbent assay (ELISA) kits from eBioscience (San Diego, CA, USA) and Invitrogen following the manufacturer's protocol.

### 2.9. Macrophage Depletion In Vivo by Clodronate-Containing Liposomes

Macrophages in the Balb/c mice were depleted by injecting clodronate-containing liposomes purchased from FormuMax Scientific Inc. (Sunnyvale, CA, USA). A starting dose of 200 *μ*L clodronate-containing liposomes for a mouse body weight of 20–25 g was intraperitoneally injected into Balb/c mice 2 d before the start of the study using imiquimod (IMQ). To prevent repopulation of macrophages, the first injection was followed by repeated injections of 100 *μ*L clodronate-containing liposomes every fourth day.

### 2.10. Flow Cytometric Analysis

For analysis of macrophage depletion efficiency, whole blood samples were collected from PBS and clodronate-containing liposome-treated mice and red blood cells (RBC) were lysed by RBC Lysis Buffer (Thermo Fisher Scientific, Invitrogen). After RBC lysis, cells were washed twice by 1x PBS. These cells were suspended in PBS containing 2% FCS and incubated with PE-conjugated F4/80 (eBioscience) and APC-conjugated CD45 (eBioscience) at 4°C for 30 min. For analysis of M1 and M2 distribution in psoriatic lesions, same sizes of skin tissues from mice were harvested and digested. Cells were counted and then incubated with PE-F4/80 (eBioscience), APC-CD86 (eBioscience), and FITC-CD206 (BioLegend) at 4°C for 30 min. After washing, cells were analyzed on a FACSCalibur flow cytometer with CellQuest software (Becton Dickinson, San Jose, CA).

### 2.11. Animal Model of Psoriatic Inflammation

In this model, 62.5 mg of 5% IMQ gel (Aldara™) was smeared on the shaved backs of Balb/c mice each day for 5 days. The severity of the skin's inflammatory response was assessed on the basis of the Psoriasis Area Severity Index (PASI) as described [34]. Briefly, the three parameters of psoriasis responses—erythema, scaling, and skin thickness—were scored independently on a scale from 0 to 4 as follows: 0: none; 1: slight; 2: moderate; 3: marked; and 4: very marked. By adding the scores from these three parameters, the severity of the response was measured using the cumulative score from 0 to 12. After 5 d of IMQ treatment, the mice were sacrificed for a more accurate measurement of skin thickness with vernier caliper.

### 2.12. Statistical Analyses

All data are presented as the means ± SD. Statistical analyses were performed on the data from three or more independent experiments using Student's *t*-test. A *P* value < 0.05 was considered to be a statistically significant difference among the experimental groups.

## 3. Results

### 3.1. Accumulation of Macrophages and Inflammation in the Psoriatic Lesions of Patients

**Figure 1:**
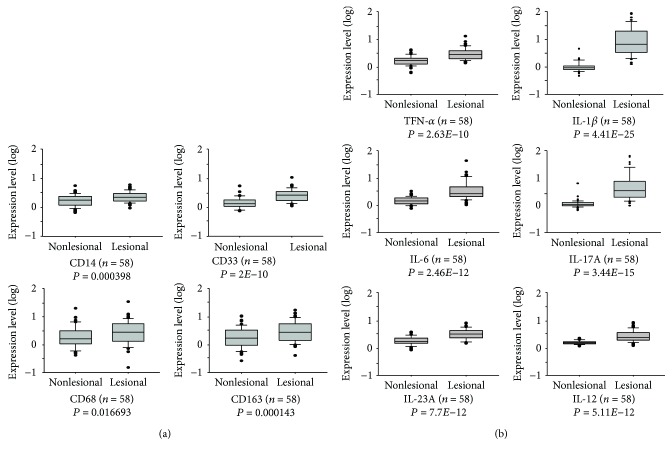
Elevated expression of monocytes and macrophage markers and inflammatory cytokines in human psoriatic lesions. Gene expression data in Gene Expression Omnibus (GEO) dataset GSE13355 were analyzed for (a) expression of monocyte and/or macrophage markers and (b) inflammatory cytokines in tissue with and without lesions from psoriatic patients (*n* = 58).

To investigate the role of macrophages in endosomal TLR-activated psoriatic inflammation, we first investigated the expression of macrophage markers and inflammatory cytokines in psoriatic lesions. A microarray dataset with data on gene expression in normal and lesional tissue samples from 58 patients with psoriasis was identified from the GEO database (GSE13355). This dataset was deposited by the Collaborative Association Study of Psoriasis (CSAP) for their genetic study to identify susceptibility factors of psoriasis [35]. The expression of monocyte and/or macrophage markers, such as cluster of differentiation 14 (CD14), CD33, CD68, and CD163, and the expression of inflammatory cytokines including TNF-*α*, IL-1*β*, IL-6, 12A, IL-17A, and IL-23A in these tissues were analyzed. The results indicated a significantly increased expression of these markers and cytokines in psoriatic tissues over those in normal tissues (Figures 1(a) and 1(b)), suggesting an associated inflammation and accumulation of monocytes and macrophages in the psoriatic lesions.

### 3.2. Involvement of Macrophages and Macrophage Polarization in Imiquimod-Activated Psoriatic Inflammation

**Figure 2:**
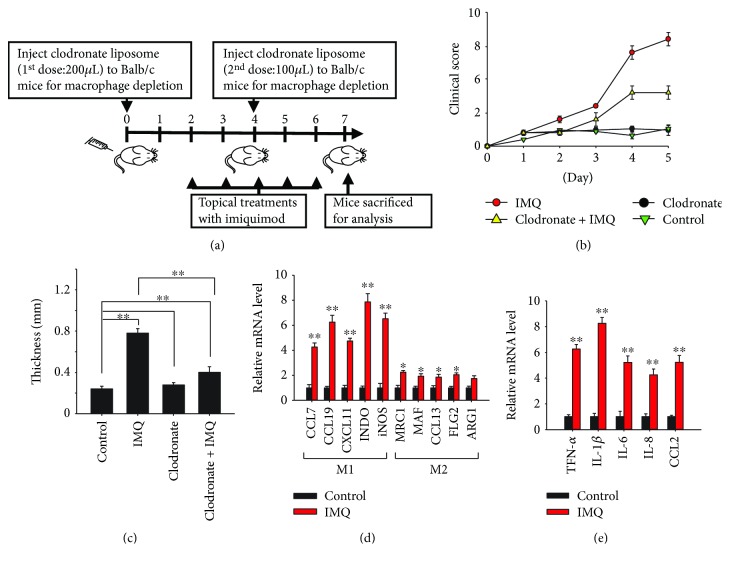
Depletion of macrophage attenuates imiquimod- (IMQ-) induced psoriasis-like inflammation. (a) Balb/c mice were intraperitoneally injected with clodronate-containing liposomes and topically treated with IMQ cream following the schedule illustrated. (b) The severity of inflammatory responses on the skin was assessed on the basis of the Psoriasis Area Severity Index. (c) Five days after IMQ treatment, the mice were sacrificed and their skin thickness was measured with vernier caliper to assess the severity of the psoriatic responses. (d, e) Tissue samples from the control and IMQ-treated mice were analyzed using quantitative real-time polymerase chain reaction for the expression of (d) M1 and M2 macrophage markers and (e) inflammatory cytokines and chemokines. The data represent mean ± standard deviation (*n* = 5); ^∗^ *P* < 0.05 and ^∗∗^ *P* < 0.01 compared with the controls, or as indicated.

**Figure 3:**
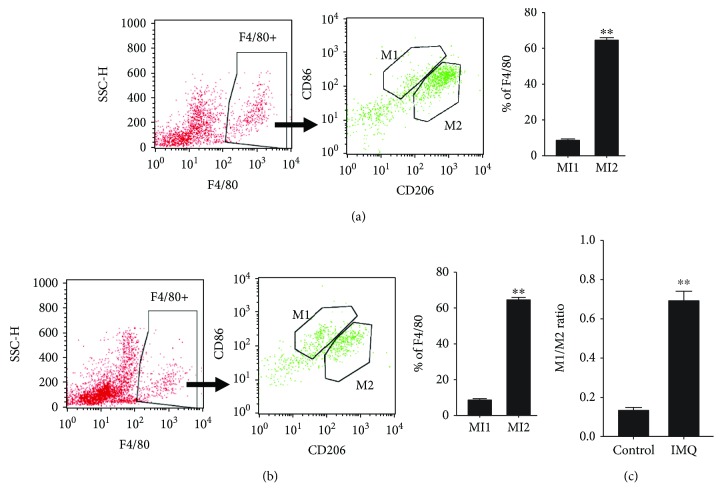
Increased M1/M2 macrophage ratio in the imiquimod-induced psoriatic lesions. Balb/c mice were treated with imiquimod (IMQ) for 5 days as illustrated in Figure 2(a). Macrophages in the tissues were analyzed by flow cytometer. (a, b) A set of representative histograms is shown for the gated population of F4/80-positive macrophages, F4/80 CD86 double-positive M1 macrophages, and F4/80 CD206 double-positive M2 macrophages in tissues from (a) control and (b) IMQ-treated mice. (c) The ratio of M1 and M2 macrophages in tissues from control- and IMO-treated mice was calculated from the bar figures in (a) and (b). Bar figures: the data represent mean ± standard deviation (*n* = 5), ^∗∗^ *P* < 0.01.

Imiquimod (IMQ) is an agonist of TLR 7, which is a member of endosomal TLRs and closely related to TLR 8 and TLR 9 [5]. Aldara™ is a 5% IMQ cream that is approved for the treatment of superficial basal cell carcinoma and genital warts [36, 37]. Topical treatment with Aldara™ on the shaved mouse back caused inflammation that closely resembled symptoms of human psoriasis, including skin thickening, scaling, and erythema [34, 38–40]. We used this animal model of IMQ-induced psoriasis in macrophage-depleted mice to investigate the role of macrophages and their polarization in endosomal TLR-mediated psoriatic inflammation. Injection of clodronate-containing liposomes into Balb/c mouse was able to deplete about two-third of the macrophages in mouse (Supplementary Figure S1). As illustrated in Figure 2(a), the mice were subcutaneously injected with clodronate-containing liposomes every 4 d to deplete their macrophages; the remaining mice were injected with a control vehicle. The injected mice were treated daily for 5 days with the 5% IMQ cream to activate psoriatic responses. The severity of the IMQ-induced psoriatic inflammatory response on the mouse skin was evaluated using the PASI score. IMQ induced a psoriatic response in mice, and the PASI scores were lower in the macrophage-depleted mice (Figure 2(b)). After 5 d of IMQ treatment, the mice were sacrificed to obtain an accurate measurement of skin thickness after the psoriatic responses. The results showed that depleting the macrophages in the mice reduced the skin thickness that was increased by the IMQ treatment (Figure 2(c)). These results suggested that macrophages play a role in mediating psoriatic inflammation activated by TLR 7. The phenotype of the macrophages in the psoriatic lesions that resulted from IMQ treatment was further investigated. Expression of M1 macrophage markers, including chemokine (C-C motif) ligand 7 (CCL7), CCL19, CXCL11, indoleamine 2,3-dioxygenase (INDO), and inducible nitric oxide synthase (iNOS), and of M2 macrophage markers, including mannose receptor C-type 1 (MRC1), MAF bZIP transcription factor (MAF), CCL13, filaggrin family member 2 (FLG2), and arginase 1 (ARG1) [41, 42], was analyzed using RT-qPCR. There was a higher expression of M1 macrophage markers than M2 macrophage markers in the psoriatic tissues (Figure 2(d)). In addition, expression of cytokine genes, including TNF-*α*, IL-1*β*, IL-6, IL8, and CCL2, which are signatures of M1 macrophages, increased in IMQ-induced psoriatic tissues (Figure 2(e)). In line with these, analysis with flow cytometry for the F4/80 CD86 double-positive M1 macrophages and the F4/80 CD206 double-positive M2 macrophages revealed a shift from a lower M1/M2 macrophage ratio in the tissues from control mice to a higher M1/M2 macrophage ratio in the tissues from imiquimod-treated mice (Figure 3). CD86 and CD206 are commonly used as cell surface markers for M1 and M2 macrophages, respectively [43].

### 3.3. Induction of M1 Macrophage Polarization by TLR 7–9 Ligands

**Figure 4:**
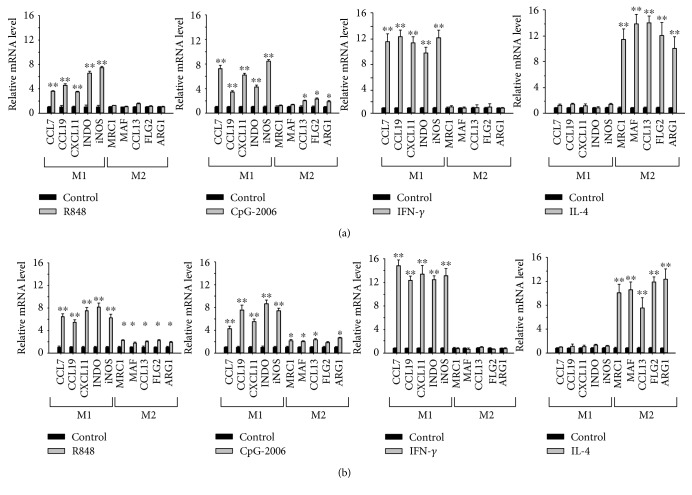
Induction of M1 macrophage polarization by endosomal Toll-like receptor (TLR) 7–9 ligands. (a) THP-1 macrophages and (b) bone marrow-derived macrophages were treated with 2 *μ*M R848 or CpG-2006 for 24 h. In addition, these cells were treated with 20 ng/mL interferon- (IFN-) *γ* and interleukin- (IL-) 4 for 24 h for control of M1 and M2 macrophage polarization, respectively. Polarization of the macrophages into M1 and M2 phenotypes was determined by quantitative real-time polymerase chain reaction analysis for expression of their signature genes. Data represent mean ± standard deviation of three independent experiments; ^∗^ *P* < 0.05 and ^∗∗^ *P* < 0.01 compared with the controls.

To determine whether TLR 7–9 activation results in macrophage polarization into M1 phenotypes, human PMA-activated THP-1 macrophages and mouse BMDMs were treated with different TLR ligands. R848 was used to activate both TLR 7 and TLR 8 in human cells. The mouse TLR 8 has very low activity [44]; therefore, the effect of R848 in mice is mostly generated from activation of TLR 7. CpG-2006 is the ligand of TLR 9 in both human and mouse cells. Similar to the effect of IFN-*γ*, a known inducer of M1 macrophage polarization, but in contrast to IL-4, a known inducer of M2 macrophage polarization, the ligands for TLRs 7–9 induced the M1 polarization of THP-1 macrophages (Figure 4(a)) and BMDMs (Figure 4(b)). Notably, the TLR 7–9 ligands induced a lower expression of M1 markers in cells than that induced by IFN-*γ*. Moreover, although the majority of M1 markers was induced, a small but significant increase in M2 markers of cells in response to the TLR 7–9 ligand stimulus was observed (Figure 4). These results are consistent with the diverse and plastic features of macrophages. Macrophages continue to change even after adopting a phenotype [23]. The IFN-*γ*- and IL-4-polarized M1 and M2 macrophages were further examined with ELISA for their cytokine production profiles. Consistent with their phenotype, the IFN-*γ*-polarized macrophages produced higher amounts of TNF-*α*, IL-1*β*, and IL-6 but a lower amount of IL-10. In contrast, the IL-4-polarized macrophages produced a higher amount of IL-10 but lower amounts of TNF-*α*, IL-1*β*, and IL-6 (Supplementary Figure S2). For comparison, the effect of other TLR ligands on macrophage polarization was also investigated. The TLR 2 ligand Pam3Cys, TLR 3 ligand polyI:C, and TLR 4 ligand LPS also induced M1 polarization of THP-1 macrophages and BMDM cells (Supplementary Figure S3).

### 3.4. Induction of M1 Macrophage Polarization and Cytokine Production by Endogenous TLR 7–9 Ligands

**Figure 5:**
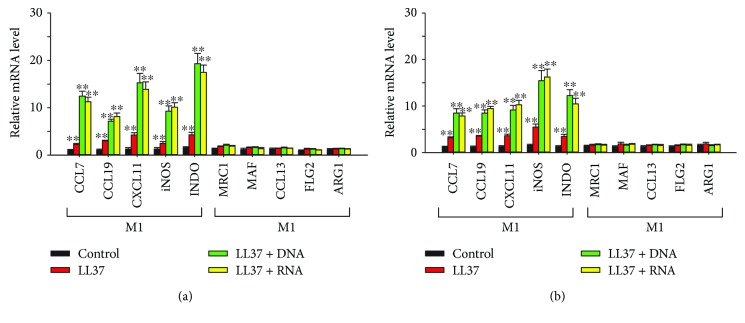
Induction of M1 macrophage polarization by endogenous Toll-like receptor (TLR) 7–9 ligands. (a) THP-1 macrophages and (b) bone marrow-derived macrophages were treated with 2 *μ*g/mL LL37, LL37/DNA, or LL37/RNA complex for 24 h. Polarization of the macrophages into M1 and M2 phenotypes was determined by quantitative real-time polymerase chain reaction (RT-qPCR) analysis for expression of their signature genes. Data represent mean ± standard deviation of three independent experiments; ^∗∗^ *P* < 0.01 compared with the controls.

**Figure 6:**
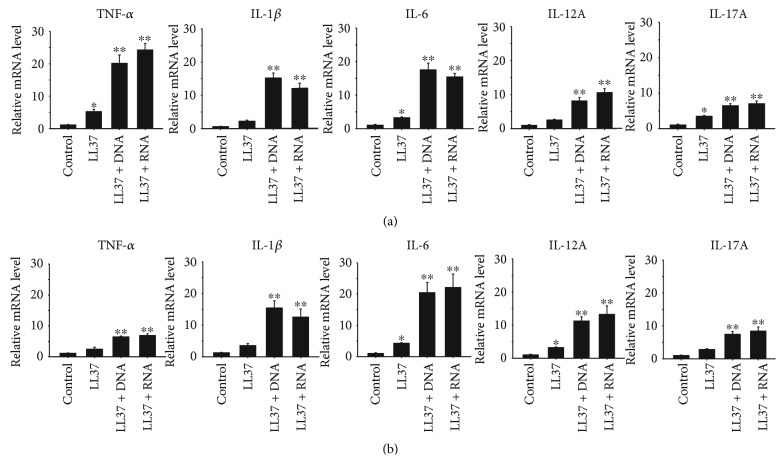
Induction of cytokine production by endogenous Toll-like receptor (TLR) 7–9 ligands. (a) THP-1 macrophages and (b) bone marrow-derived macrophages were treated with 2 *μ*g/mL LL37, LL37/DNA, or LL37/RNA complex for 24 h. Production of cytokines was determined by quantitative real-time polymerase chain reaction (RT-qPCR) analysis. Data represent mean ± standard deviation of three independent experiments; ^∗^ *P* < 0.05 and ^∗∗^ *P* < 0.01 compared with the controls.

We further investigated whether the endogenous ligands of TLRs 7–9, the LL37/DNA and LL37/RNA complexes which appear in psoriatic lesions [28–30], can induce macrophage polarization. THP-1 macrophages and BMDMs were treated with control vehicle, LL37, LL37/DNA complex, and LL37/RNA complex. RT-qPCR analysis of the expression of M1 and M2 macrophage signature genes revealed that LL37 mildly activated M1 polarization. In contrast, the LL37/DNA and LL37/RNA complexes were more potent in inducing M1 polarization of the THP-1 macrophages and BMDMs (Figures 5(a) and 5(b)). For comparison, RT-qPCR was also used to investigate the effect of these endogenous TLR 7–9 ligands on inducing macrophages to produce cytokines. The results revealed that similar to their capability to activate M1 polarization of THP-1 macrophages and BMDMs, the LL37/DNA and LL37/RNA complexes activated the expression of the proinflammatory cytokine genes TNF-*α*, IL-1*β*, IL-6, IL-12A, and IL-17A, which are signatures of M1 macrophages (Figures 6(a) and 6(b)).

### 3.5. M1 Macrophages Contain Higher Expression Levels of Inflammatory Cytokines and TLRs 7–9 than M2 Macrophages

**Figure 7:**
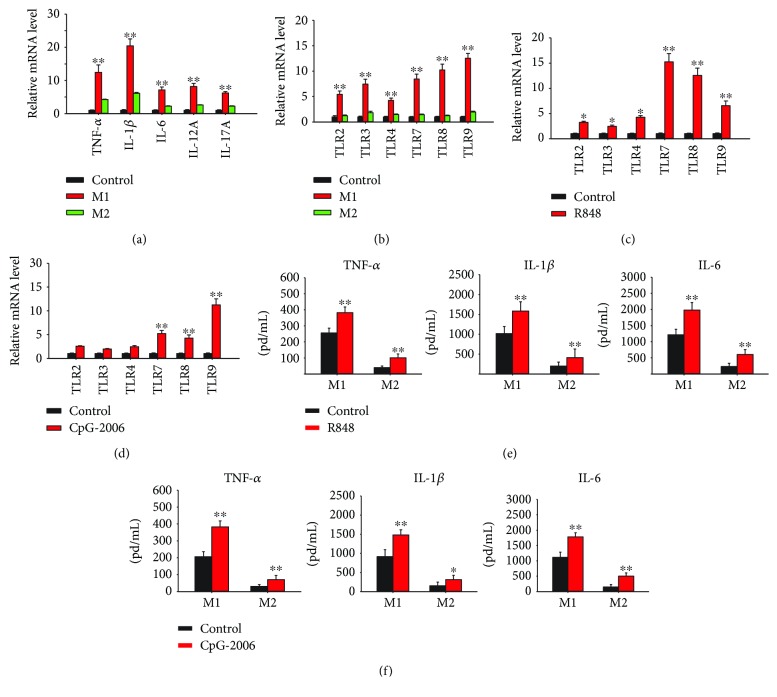
High expression of proinflammatory cytokines and Toll-like receptors (TLRs) in M1 macrophages and induction of TLR 7–9 expression by their agonists in macrophages. THP-1 macrophages were treated with 20 ng/mL interferon- (IFN-) *γ* and interleukin- (IL-) 4 to polarize them into M1 and M2 macrophages. (a) Expression of genes for proinflammatory cytokines and (b) expression of TLRs in these cells were analyzed using quantitative real-time polymerase chain reaction (RT-qPCR). (c, d) To assess the capability of inducing the expression of TLRs 7–9 by their agonists, THP-1 macrophages were treated with 2 *μ*M (c) R848 or (d) CpG-2006 for 24 h and expression of different TLRs was analyzed using RT-qPCR. (e, f) The IFN-*γ*- and IL-4-polarized M1 and M2 macrophages were treated with 2 *μ*M (e) R848 or (f) CpG-2006 for 24 h, and production of cytokines as indicated in medium was measured with enzyme-linked immunosorbent assay. Data represent mean ± standard deviation of three independent experiments; ^∗^ *P* < 0.05 and ^∗∗^ *P* < 0.01 compared with the controls (c–f) or between M1 and M2 macrophages (a, b).

To evaluate the role of M1 macrophage polarization on mediating psoriatic inflammation, the expression levels of the inflammatory cytokine genes and various TLR genes in both the M1 and M2 macrophages were further compared. THP-1 macrophages were polarized into M1 and M2 phenotypes after treating with IFN-*γ* and IL-4, respectively. RT-qPCR was conducted to analyze the expression of different inflammatory cytokines, such as TNF-*α*, IL-1*β*, IL-6, IL-12A, and IL-17A, in these macrophages. The results showed a higher expression of proinflammatory cytokines in the M1 than M2 macrophages (Figure 7(a)). In addition, RT-qPCR analysis revealed a higher expression of different TLRs, including TLRs 7–9, in the M1 macrophages than in the M2 macrophages (Figure 7(b)). The expression levels of different TLRs in R848- and CpG-2006-activated macrophages were also investigated. The macrophages activated by the TLR 7–9 ligands contained higher expression levels of various TLRs, including TLRs 7–9, than the untreated THP-1 macrophages (Figures 7(c) and 7(d)). The IFN-*γ*- and IL-4-polarized M1 and M2 macrophages were stimulated with R848 and CpG-2006 and analyzed by ELISA for the production of inflammatory cytokines, including TNF-*α*, IL-1*β*, and IL-6. The results showed higher basal production of inflammatory cytokines in M1 macrophages, and stimulation by R848 and CpG-2006 further increased the cytokine levels (Figures 7(e) and 7(f)). These results indicated that the M1 macrophages are more inflammatory and have a capacity to further sense TLR 7–9 ligands following polarization by TLR 7–9 activation at the psoriatic sites.

### 3.6. Using Inhibitors to Block TLR 7- to TLR 9-Activated M1 Macrophage Polarization and Cytokine Production

**Figure 8:**
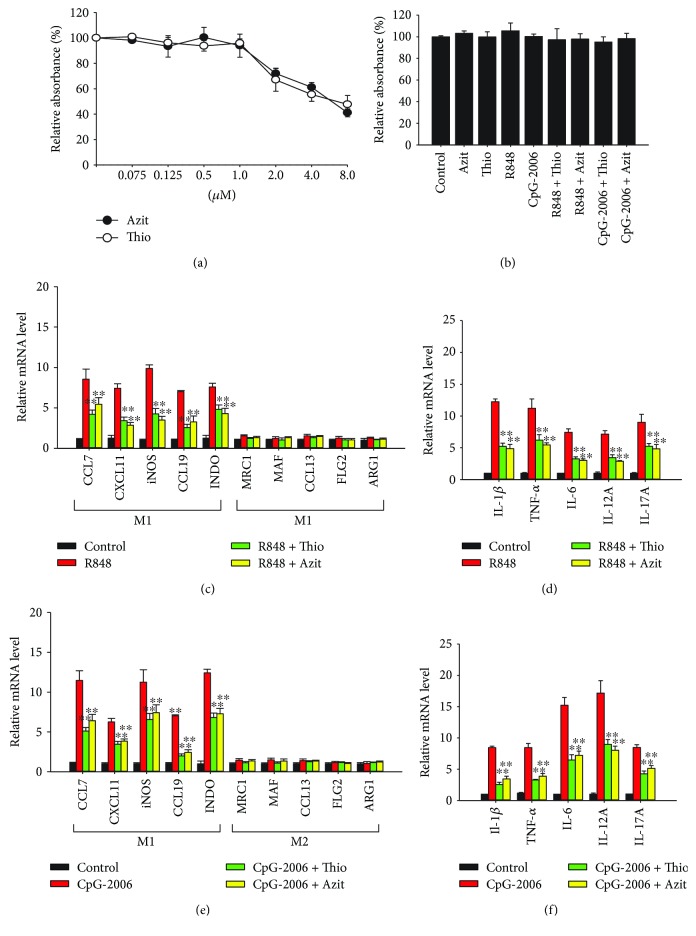
Thiostrepton and azithromycin attenuate Toll-like receptor (TLR) 7- to 9-induced M1 macrophage polarization and cytokine production *in vitro* and *in vivo*. (a, b) Bone marrow-derived macrophages (BMDMs) were treated with different concentrations of (a) thiostrepton (Thio) and azithromycin (Azit) and (b) 1 *μ*M Thio or Azit with or without 2 *μ*M R848 or CpG-2006 to assess the cytotoxicity of these treatments. (c–f) The cells were treated with 1 *μ*M Thio or Azit plus 2 *μ*M (c, d) R848 or (e, f) CpG-2006 for 24 h. Expression of signature genes for (c, e) M1 and M2 macrophages and expression of genes for (d, f) inflammatory cytokines were analyzed with quantitative real-time polymerase chain reaction (RT-qPCR). Data represent mean ± standard deviation of three independent experiments; ^∗∗^ *P* < 0.01 compared with the R848- or CpG-2006-treated group (c–f).

Different TLR 7–9 inhibitors, such as thiostrepton and azithromycin, blocked cytokine production induced by TLR 7–9 ligands and reduce psoriatic inflammation. Their functional mechanisms involve blocking endosomal acidification, inhibiting proteases, and trafficking TLRs 7–9 to endosomes for proper function [38, 45]. The effect of these inhibitors on TLRs 7–9 ligand-induced M1 macrophage polarization has not been investigated. To study the effects of thiostrepton and azithromycin, we first investigated the cytotoxicity of these two compounds on BMDMs. The cells were treated with different concentrations of the two inhibitors, and MTS assays were conducted to analyze cell viability. The results showed that BMDMs were resistant to the two inhibitors up to a concentration of 1 *μ*M (Figure 8(a)). Furthermore, the cells were also resistant to different combinations of 1 *μ*M thiostrepton or azithromycin with 2 *μ*M R848 or CpG-2006 (Figure 8(b)). Thus, BMDMs were treated with TLR 7–9 ligands at a concentration of 2 *μ*M in the presence and absence of 1 *μ*M thiostrepton and azithromycin and cytokine production and macrophage polarization were analyzed. The results indicated that both thiostrepton and azithromycin inhibited R848- and CpG-2006-activated M1 macrophage polarization (Figures 8(c) and 8(e) **)**. In addition, these two inhibitors blocked TLR 7- to TLR 9-induced expression of cytokine genes (Figures 8(d) and 8(f)).

### 3.7. Role of M1 Macrophage Polarization in Endosomal Toll-Like Receptor-Activated Psoriatic Inflammation

**Figure 9:**
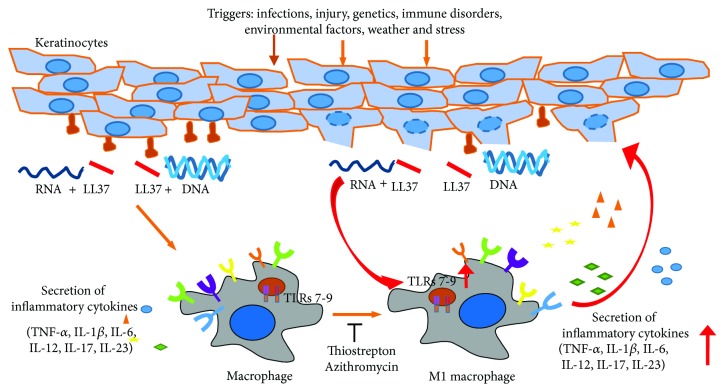
Model for the role of macrophages in the pathogenic role of Toll-like receptor (TLR) 7- to 9-activated psoriatic inflammation. Ligands of TLRs 7–9 activate cytokine production, TLR 7–9 expression, and M1 polarization in macrophages. M1 macrophages express higher levels of proinflammatory cytokines and TLRs 7–9. These render macrophages to be more inflammatory and further respond to the TLR ligands and lead to an amplification of TLR 7- to 9-activated inflammation at the psoriatic sites. Inhibitors of TLRs 7–9 such as thiostrepton and azithromycin block this TLR-activated M1 macrophage polarization, which can be a mechanism for their inhibitory activity in reducing psoriatic inflammation. Red arrows show the increased expression of TLRs 7–9 and proinflammatory cytokines.

Overall, as shown in Figure 9, the present study suggested that polarization of the M1 macrophage activated by TLRs 7–9 plays a role in the pathogenic activity of the endosomal TLRs in psoriasis. The M1 macrophages are more inflammatory and are capable to sense TLR 7–9 ligands. Thus, this endosomal TLR-activated inflammation at the psoriatic sites can be amplified by inducing macrophages into the M1 phenotype. In addition, blocking M1 macrophage polarization using thiostrepton and azithromycin is part of their functional mechanism to reduce endosomal TLR-activated psoriatic inflammation.

## 4. Discussion

In the present study, we investigated a macrophage-involved mechanism to determine the pathogenic role of TLRs 7–9 in psoriatic inflammation. These three endosomal TLRs have been shown to be involved in the pathogenesis of psoriasis. This is evidenced by the induction of psoriatic responses by IMQ gel, which contains a TLR 7 agonist [34, 38–40]. As shown in the present and previous studies, consecutive treatment with IMQ gel on the ear or shaved backs of mice causes inflammation that closely resembled symptoms of human psoriasis, such as increased skin thickness, scaling, and erythema [34, 38–40]. In addition, psoriasis has been associated with the clinical application of IMQ gel in patients with basal cell carcinoma or actinic keratosis [46–48]. Nevertheless, a variety of results has been reported about the contribution of TLR 7 to the psoriatic responses induced by the IMQ gel. Walter et al. used TLR 7 knockout mice to show that the psoriatic responses induced by the Aldara/IMQ gel were largely TLR 7 independent [49]. In contrast, Ueyama et al. showed that the psoriatic inflammatory effects induced by Beselna/IMQ gel, which contains the same composition as Aldara gel, are mediated by TLR 7 in TLR 7 knockout mice [50]. Similarly, other reports have revealed the resistance of MyD88 knockout mice to Aldara/IMQ-induced psoriatic skin inflammation [51, 52]. A wide variety of inhibitors of these endosomal TLRs, including thiostrepton and azithromycin, attenuates IMQ-induced psoriatic responses in animal models [17, 38, 45]. Furthermore, direct targeting of TLRs 7–9 has also been investigated as a potential therapy to treat psoriasis. In a phase 2 clinical trial study with psoriasis patients, immune modulatory oligonucleotide- (IMO-) 3100, an antagonist of TLRs 7 and 9, was shown to reduce the PASI score. Similarly, IMO-8400, a second-generation IMO that antagonizes TLRs 7–9, was demonstrated to have clinical activity in a phase 2a clinical study of patients with moderate-to-severe plaque psoriasis [53, 54]. These findings further support the pathogenic role of these TLRs in psoriasis.

The underlying mechanism of TLR 7- to TLR 9-activated psoriasis was investigated; however, more focus was on their activation in DCs because these cells are highly expressed with TLRs. Plasmacytoid DCs express TLRs 7 and 9, and myeloid DCs express TLRs 7 and 8 [55]. Activation of TLRs 7–9 in DCs by their cognate ligands, including the LL37/DNA and LL37/RNA complexes, resulted in cytokine production. These cytokines further activated inflammatory responses in psoriatic lesions, including differentiation of T cells into different subtypes for further production of different cytokines, proliferation of keratinocytes, and the recruitment of inflammatory cells, such as neutrophils and macrophages, into the psoriatic lesions [31–33]. In contrast, the role of macrophages in mediating endosomal TLR-involved pathogenesis of psoriasis has not been investigated [31–33]. Macrophage is a type of cell that plays a critical role in inflammatory responses. For example, macrophages are the major source of TNF-*α* in psoriatic lesions and anti-TNF-*α* agents are approved for treatment of psoriasis [56]. Monocytes and macrophages are constitutively expressed with TLRs 7–9. Moreover, the expression of these TLRs increases in response to inflammatory and microbial stimuli [57, 58]. The increased expression of these endosomal TLRs has been consistently detected in the mononuclear cells in the peripheral blood of patients with psoriasis [59]. This supports that macrophages might play a role in endosomal TLR-mediated psoriatic inflammation.

Macrophages can be polarized into two major differential phenotypes—M1 and M2. M1 macrophages produce proinflammatory cytokines and are associated with tissue damage; M2 macrophages generate anti-inflammatory cytokines and are thought to improve tissue repair after inflammation or injury [20–24]. A balance between these two types of macrophages can affect the outcome of inflammatory diseases [24–27]. For example, it has been shown that a decrease in M1 macrophages in CXCR1-deficient mice is associated with attenuated IMQ-induced psoriatic inflammation [60]. IL-35 decelerates psoriatic inflammation by reducing the total number of macrophages and the ratio of M1/M2 macrophages [61]. In addition, naringenin, a flavonoid compound, has been shown in a mouse model to ameliorate skin inflammation by accelerating the reprogramming of macrophages from the M1 to the M2 phenotype [62].

Consistent with these previous studies [60–62], by analysis of clinical data from patients in GEO database, our current study shows higher expression levels of monocyte and macrophage markers in association with higher expression of inflammatory cytokines in psoriatic tissues than in normal tissues. Depletion of macrophages in mice results in a reduction of IMQ-induced psoriatic inflammatory responses. These suggest that macrophages play a role in endosomal TLR-induced psoriatic inflammation. Furthermore, TLR 7–9 ligands, such as R848 and CpG-2006, and the LL37/RNA and LL37/DNA complexes activate cytokine production and M1 polarization in macrophages. M1 macrophages expressed higher levels of proinflammatory cytokines than M2 macrophages. In addition, there was higher expression of TLRs 7–9 in M1 macrophages. As shown in Figure 9, these suggested that by inducing M1 polarization, the TLR 7–9 ligands render macrophages to be more inflammatory and more susceptible to their ligands in the psoriatic sites, which could result in an amplified inflammatory response at these sites.

## 5. Conclusions

In summary, this study identified a macrophage-involved mechanism for the pathogenic role of endosomal TLRs in psoriasis and suggests that blocking macrophage polarization into the M1 phenotype could be a strategy which enables inhibition of endosomal TLR-activated psoriatic inflammation. In addition, this study shows an inhibitory effect of thiostrepton and azithromycin on M1 macrophage polarization induced by the TLR 7–9 ligands, which suggests that the previously identified inhibitory activities of these two compounds on endosomal TLR-activated psoriatic responses [38, 45] could also be partially involved with their capacity to block TLR ligand-induced M1 macrophage polarization.

## Data Availability

The data used to support the findings of this study are available from the corresponding author upon request.

## Supplementary Materials

Supplementary Materials Supplementary Table S1: nucleotide sequences of primers used for quantitative real-time polymerase chain reaction (RT-qPCR) of human genes. Supplementary Table S2: nucleotide sequences of primers used for quantitative real-time polymerase chain reaction (RT-qPCR) of mouse genes. Supplementary Figure S1: efficiency of clodronate-containing liposomes in the depletion of mouse macrophages. Supplementary Figure S2: cytokine production profiles of interferon-*γ*- and interleukin-4-polarized macrophages. Supplementary Figure S3: induction of M1 macrophage polarization by different Toll-like receptor (TLR) ligands.
