# Supplementary material for: Involvement of M1 Macrophage Polarization in Endosomal Toll-Like Receptors Activated Psoriatic Inflammation
Source: Mediators Inflamm. 2018 Dec 16;2018:3523642. doi: 10.1155/2018/3523642 (PMC6311781; doi:10.1155/2018/3523642)
Supplement: Supplementary Materials — Supplementary Table S1: nucleotide sequences of primers used for quantitative real-time polymerase chain reaction (RT-qPCR) of human genes. Supplementary Table S2: nucleotide sequences of primers used for quantitative real-time polymerase chain reaction (RT-qPCR) of mouse genes. Supplementary Figure S1: efficiency of clodronate-containing liposomes in the depletion of mouse macrophages. Supplementary Figure S2: cytokine production profiles of interferon-γ- and interleukin-4-polarized macrophages. Supplementary Figure S3: induction of M1 macrophage polarization by different Toll-like receptor (TLR) ligands. [file 3523642.f1.pdf]

Supplementary Materials

Involvement of M1 Macrophage Polarization in Endosomal Toll-like Receptors Activated Psoriatic Inflammation

Chih-Hao Lu,[1,2] Chao-Yang Lai,[1] Da-Wei Yeh,[1] Yi-Ling Liu,[1] Yu-Wen Su,[1] Li-Chung Hsu,[3] Chung-Hsing Chang,[4] S.-L. Catherine Jin,[2] and Tsung-Hsien Chuang[1, 5]

[1] *Immunology Research Center, National Health Research Institutes, Miaoli, Taiwan*
[2] *Department of Life Sciences, National Central University, Zhongli District, Taoyuan City, Taiwan*
[3] *Institute of Molecular Medicine, College of Medicine, National Taiwan University, Taipei, Taiwan*
[4] *Skin Institute, Hualien Tzu Chi Hospital, Institute of Medical Sciences, Tzu Chi University, Hualien, Taiwan*
[5] *Program in Environmental and Occupational Medicine, Kaohsiung Medical University Kaohsiung, Taiwan*

Correspondence should be address to Tsung-Hsien Chuang; E-mail: thchuang@nhri.org.tw
Running title: M1 macrophage polarization and psoriasis

Supplementary Table 1: Nucleotide sequences of primers used for quantitative real-time polymerase chain reaction (RT-qPCR) of human genes.

Supplementary Table 2: Nucleotide sequences of primers used for quantitative real-time polymerase chain reaction (RT-qPCR) of mouse genes.

Supplementary Figure 1: Efficiency of clodronate-containing liposomes in the depletion of mouse macrophages. Balb/c mice were injected with 200 μl of clodronate-containing liposomes or PBS. In the next day, the population of macrophages in blood cells was analyzed by flow cytometry. (a) The histograms shown are representative of three independent experiments. (b) Bar figure for the histograms, the data represent mean ± standard deviation (n = 3), **P < 0.01 compared with the PBS controls.

Supplementary Figure 2: Cytokine production profiles of interferin-γ and interleukin-4 polarized macrophages. THP-1 macrophages polarized treated with 20 ng/mL interferon (IFN)-γ or interleukin (IL)-4 for 24 h for M1 and M2 macrophage polarization. These cells were washed extensively and incubated for 24 h. Cytokines secreted into culture media were analyzed with Enzyme-Linked ImmunoSorbent Assay. Data represent mean ± standard deviation of three independent experiments, **$P < .01$ compared between the M1 and M2 macrophages.

Supplementary Figure 3: Induction of M1 macrophage polarization by different toll-like receptor (TLR) ligands. (a) THP-1 macrophages and (b) bone marrow-derived macrophages were treated with different TLR ligands (0.2 μg/mL Pam3Cys [TLR 2], 5 μg/mL of PolyI:C [TLR 3], and 0.2 μg/mL of LPS [TLR 4]) for 24 h. Expression of the signature genes for M1 and M2 macrophages were analyzed by quantitative real-time polymerase chain reaction (RT-qPCR). Data represent mean ± standard deviation of three independent experiments, *P < .05, **$P < .01$ compared with the controls.

Supplementary Table 1

Human	Forward	Reverse
IL-1β	ACGATGCACCTGTACGATCA	TCTTTCAACACGCAGGACAG
IL-6	TACCCCCAGGAGAAGATTCC	TTTTCTGCCAGTGCCTCTTT
IL-12A	ACTAGAGAGACTTCTTCCACAACAAGAG	GCACAGGGTCATCATCAAAGAC
IL-17A	ATGAACTCTGTCCCCATCCA	TTGAAGGATGAGGGTTCCTG
CCL7	ACATCGGAGACAACACCACA	GGAAGGGTCAGGAGGAAGAG
CCL13	CTCCTCTGGCCTCCTCTTCT	ACCGAATACAAACCCACTGC
CCL19	GGTGCCTGCTGTAGTGTTCA	GGTCCTTCCTTCTGGTCCTC
TLR2	ACTTCATTCCTGGCAAGTGG	TTTTTCTCAATGGGCTCCAG
TLR3	AGCCTTCAACGACTGATGCT	TTCCAGAGCCGTGCTAAGTT
TLR4	TTGGGACAACCAGCCTAAAG	TGCCATTGAAAGCAACTCTG
TLR7	AATGTCACAGCCGTCCCTAC	TTATTTTTACACGGCGCACA
TLR8	TGTGATGGTGGTGCTTCAAT	TCGTTAAAAATGCCCCAGAG
TLR9	AAAGAGGAAGGGGTGAAGGA	ACAGCAGCTACAGGGAAGGA
TNF-α	AACCTCCTCTCTGCCATCAA	CCAAAGTAGACCTGCCCAGA
CXCL11	TCGAAGCAAGCAAGGCTTAT	GTCCTTTCACCCACCTTTCA
GADPH	GAGTCAACGGATTTGGTCGT	GACAAGCTTCCCGTTCTCAG
ARGINASE 1(ARG1)	GGCTGGTCTGCTTGAGAAAC	TTCCCACAGACCTTGGATTC
FLG2	GGCCACAAAATGCTTCAAGT	AGGTTGACCACATCCAGAGG
NOS2(INOS)	ACAAGCCTACCCCTCCAGAT	TCCCGTCAGTTGGTAGGTTC
MRC1	TGACACACTTTTGGGGATCA	AAACTTGAACGGGAATGCAC
MAF	AGAGACACGTCCTGGAGTCG	GCTTCCAAAATGTGGCGTAT

Supplementary table 2

Mouse	Forward	Reverse
IL-1β	CAGGCAGGCAGTATCACTCA	AGCTCATATGGGTCCGACAG
IL-6	AGTTGCCTTCTTGGGACTGA	TCCACGATTTCCCAGAGAAC
IL-8	CGTCCCTGTGACACTCAAGA	TAATTGGGCCAACAGTAGCC
IL-12A	CTCCTGTGGGAGAAGCAGAC	CAGATAGCCCATCACCCTGT
IL-17A	TCCAGAAGGCCCTCAGACTA	ACACCCACCAGCATCTTCTC
CCL2	CAGGTCCCTGTCATGCTTCT	TCTGGACCCATTCCTTCTTG
CCL7	TGTACGAGTCGGTGTGCTTC	TAGGCCCAGAAGGGAAGAAT
CCL19	TTCCCAGCGGATTTTAAGTG	GCAAAAGAGGCAGACAGACC
CCL22	CCTTGTTTTGATGCCCTGAT	CCTTGTTTTGATGCCCTGAT
CCL13	ACAGTGGAGAAGAGGGAGCA	GGCCACGACTTCTCAGACTC
FLG2	GCAACAAGGTTCTTGGGAAA	CATGCTCCTCTCCCTCACTC
TNF-α	AGCCCCCAGTCTGTATCCTT	CTCCCTTTGCAGAACTCAGG
CXCL11	CAGTGCTGGATTCAAAAGCA	AACCCCTTAGAAGGCCTCAG
GADPH	ACCCAGAAGACTGTGGATGG	CACATTGGGGGTAGGAACAC
ARGINASE 1(ARG1)	CGCCTTTCTCAAAAGGACAG	GACATCAACAAAGGCCAGGT
NOS2(INOS)	CACCTTGGAGTTCACCCAGT	ACCACTCGTACTTGGGATGC
MRC1	TGGCAAGTGTCCAGAGTCAG	TCCCTTCAACATTTCGGAAC
MAF	TCCTGAGTGGGCTTGCTAGT	AAGTTACGGGGGAATTCAGG

Supplementary Figure 1

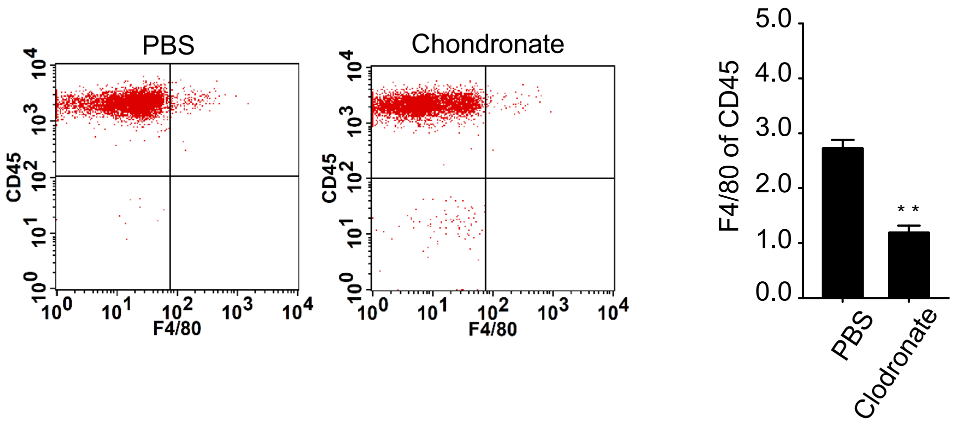

(a) (b)

Supplementary Figure 2

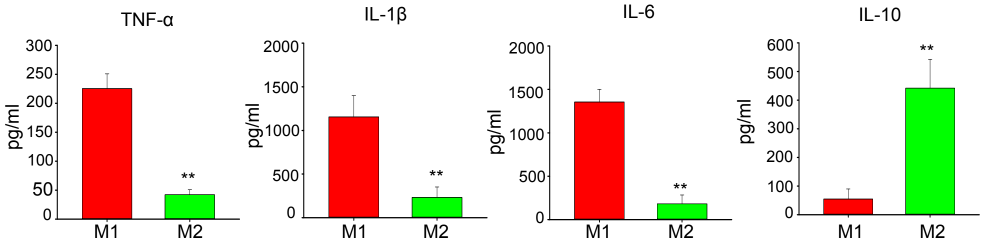

Supplementary Figure 3

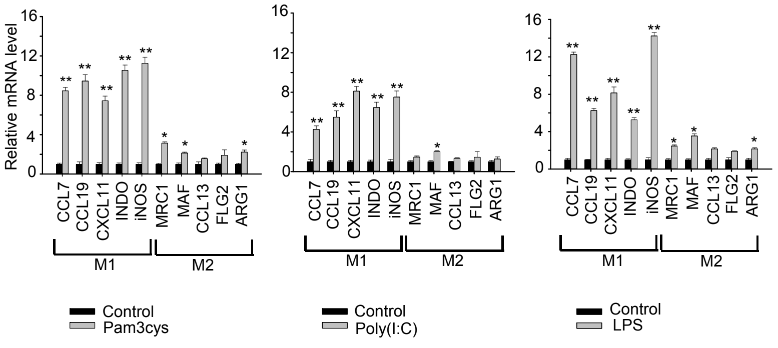

(a) THP-1

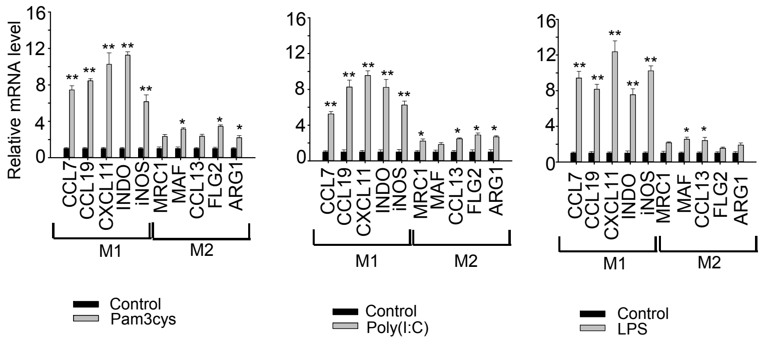

(b) BMDM
